# Birthing outside the system: the motivation behind the choice to freebirth or have a homebirth with risk factors in Australia

**DOI:** 10.1186/s12884-020-02944-6

**Published:** 2020-04-28

**Authors:** Melanie K Jackson, Virginia Schmied, Hannah G Dahlen

**Affiliations:** School of Nursing and Midwifery, Western Sydney University, Locked bag 1797, Penrith, NSW 2751 Australia

**Keywords:** Australia; freebirth, Unassisted birth, Homebirth, Midwives, High-risk, Medicalisation

## Abstract

**Background:**

Childbirth in Australia occurs predominantly in a biomedical context, with 97% of births occurring in hospital. A small percentage of women choose to *birth outside the system –* that is, to have a midwife attended homebirth with risk factors, or a freebirth, where the birth at home is intentionally unattended by any health professional.

**Method:**

This study used a Grounded Theory methodology. Data from 13 women choosing homebirth and 15 choosing freebirth were collected between 2010 and 2014 and analysed over this time.

**Results:**

The core category was ‘wanting the best and safest,’ which describes what motivated the women to *birth outside the system.* The basic social process, which explains the journey women took as they pursued the best and safest, was ‘finding a better way’. Women who gave *birth outside the system* in Australia had the countercultural belief that their knowledge about what was best and safest had greater authority than the socially accepted experts in maternity care. The women did not believe the rhetoric about the safety of hospitals and considered a biomedical approach towards birth to be the riskier birth option compared to giving birth outside the system. Previous birth experiences taught the women that hospital care was emotionally unsafe and that there was a possibility of further trauma if they returned to hospital. Giving *birth outside the system* presented the women with what they believed to be the opportunity to experience the best and safest circumstances for themselves and their babies.

**Conclusion:**

Shortfalls in the Australian maternity care system is the major contributing factor to women’s choice to give birth outside the system. Systematic improvements should prioritise humanising maternity care and the expansion of birth options which prioritise midwifery-led care for women of all risk.

## Background

The majority of births (97%) in Australia occur in hospitals, 1.8% of women give birth in birth centres and 0.3% at home [1]. Around 0.4% of babies are born before arrival (BBA) to a hospital or birth centre [1]. It is possible some of this BBA data represent planned freebirths [2], however data is not collected on freebirths in Australia and other countries [3, 4]. For the purposes of this research, a freebirth is defined as ‘a planned homebirth that the parents arrange to be intentionally unattended by any registered midwife or obstetrically trained registered professional’ [3]. A homebirth with risk factors is defined as any midwife attended planned homebirth where there are known maternal or fetal risk factors that would normally exclude a woman from a publicly funded homebirth program. In Australia publicly funded homebirth is run out of public hospitals and under State/Territory guidelines. However, privately practising midwives attend more than half the homebirths and they have more flexibility around which women they care for.

The phenomenon of women choosing to freebirth or have a homebirth with risk factors homebirth has been explored by national and international researchers [4–14]. These studies indicate that women are choosing to birth outside the system due to their rejection of biomedical models of care [4–6, 9, 11, 13, 15]. Women who chose to birth outside the system express differing beliefs about risk and safety; hospital is often seen as dangerous and home as safe [4, 5, 9, 12, 15, 16]. These women have faith in their bodies, their instincts and the process of birth as a normal life event [4, 5, 9, 15]. The desire for autonomy during pregnancy and birth [4, 9, 13, 15] and concerns about being disturbed during birth with interruptions and unnecessary interventions also fuel women’s desires to give birth outside the system [4, 10, 11].

A previous traumatic birth experience is another major driver [6, 11, 13, 17]. Women report that in the past their choices were not respected and birthing outside the system is seen as an opportunity to ensure this does not happen again [4]. Women are also choosing freebirth over midwife-attended homebirth due to difficulties accessing midwives and covering the financial cost of a care provider [13, 18]. In 2020, the authors of this paper published a book and provided international research on the question of why women are giving birth outside of the system [19]. What is clear is that solutions to the phenomena of birth outside the system are available and need to be heeded.

Globally, childbirth occurs within predominantly biomedical models of care [20, 21] and medical authorities in Australia propose that homebirth is unsafe [22], despite evidence of safety for low risk women [23–25]. Existing biomedical childbirth services are not meeting women’s needs [26] and recent Australian research suggests that there is an increase in use of unregulated birth workers as a result [13]. The decision to birth outside the system reveals tension between biomedically focused maternity care and women’s expectations [15]. It is becoming clear that a rise in the rates freebirth and homebirth with risk factors in Australia is symptomatic of inadequate maternity care options [13, 17, 26].

The aim of this study was to explore what motivates Australian women to *birth outside the system –* that is, to have a midwife attended homebirth despite identified complexities, or a freebirth where birth at home is planned to be intentionally unattended by health professionals.

## Methods

This research was conducted between 2010 and 2014. Grounded Theory was the most suitable research method for this study, because it is ideal in circumstances where relatively little is known about a subject [27]. Grounded Theory serves to answer ‘why’ questions in addition to ‘what’ questions of a phenomenon, thus resulting in the generation of new knowledge [28]. This renders it an ideal tool with which to pose the question ‘*what* motivates women to *birth outside the system*?’

Birks and Mills (2011) suggest ten essential elements that should be used in the research process if a Grounded Theory is to be produced. These are: initial coding and catergorisation of data, concurrent generation or collection of data and analysis, writing memos, theoretical sampling, constant comparative analysis, theoretical sensitivity, intermediate coding, identifying a core category, advanced coding and theoretical integration and generating a theory. The application of Birks and Mills’ (2011) suggestion of adhering to the ten essential elements was adopted in this study [28, 29]. The essential elements suggested by Birks and Mills (2011) adhere most closely to a Glaserian approach to the development of Ground Theory. From Glaser’s perspective, emergence is an essential element to ensure that the final theory is grounded in the data. Glaser’s stance is that by the application of Grounded Theory principles – such as constant comparison and coding and analysing – categories and their properties will emerge [30].

### Recruiting and selecting participants

The process of selecting research participants for Grounded Theory research relies on the use of ‘theoretical sampling,’ [28], where the researcher specifically seeks pertinent data to refine categories as the theory emerges [29]. Theoretical sampling feeds constant comparative analysis and helps to saturate categories [28].

In the beginning of the recruitment process, two women known to the authors were purposefully approached to participate in this research; one had had a freebirth and the other a birth at home with risk factors. Both consented to being interviewed and from there ‘snowball sampling’ was used, thus, study participants recommend future participants. Another valuable exercise during the recruitment process was to announce the research at the Homebirth Australia Conference in 2010 as this generated a lot of interest and many women and midwives offered contact information as potential participants. These volunteers were carefully screened against the selection criteria before being included in the study. As the study progressed, participants were theoretically sampled based on personal information they gave during recruitment. Charmaz (2006), suggests that theoretical sampling only becomes of value once your categories have been developed, as this enables the researcher to confirm and clarify these categories.

### Participants

We planned to recruit women who had experienced or were planning a freebirth or a homebirth with risk factors.

The inclusion criteria for the participants were as follows:

- Have had a freebirth or homebirth with risk factors in the past
- Are pregnant and are planning a freebirth or homebirth with risk factors
- Are intending to have a freebirth or homebirth with risk factors in the future

Participants were included if their intention was to birth outside the system, regardless if they ultimately gave birth elsewhere. The criteria were defined as such because the interest was in what motivated a woman’s choice; whether or not the woman actually achieved a *birth outside the system* was therefore irrelevant. In total, the stories of 28 women were included as data for the study, 13 who had chosen homebirth and 15 choosing freebirth.

#### Data collection and analysis

Of the 28 stories included in this research, eleven of the women choosing homebirth were interviewed, with one homebirth woman’s story sourced from the 2009 *National Review of Maternity Services in Australia* (NMR) [31], and another from a story sourced on the internet, separate from the NMR data. Nine of the women who planned freebirths were interviewed, with the remaining six freebirth stories being sourced from the NMR. The decision to use secondary data is described below.

### In-depth interviews

Participants (*n* = 20) were interviewed at a location and time of their choosing. To begin, women provided demographic information, and from there a flexible interview style was used with open-ended questions. To start the interview, the participants were invited to just tell their story about their birth choice and as they went the interviewer responded with questions to gather a deeper understanding. Many of the questions aimed to delve deeper into the motivations behind women’s choices; for example, ‘Why did you choose that particular care provider?’, ‘Can you tell me more about why you were determined not to have any intervention?’, ‘What things will you do differently this time?’. Interview questions were revised for the next participant, based on what was discovered in previous interviews. The questions continued to be broad and open-ended, aimed at discovering new information and as a means of clarifying existing data. In line with the principles of theoretical sampling, and as data analysis progressed, women whose stories related to categories that were already considered ‘saturated’ were not pursued for interview, while those who expressed a new idea were recruited. This allowed for the generation of new information. Categories were considered saturated if participants consistently said the same thing as had already been discovered.

### Use of secondary data - submissions to NMR and on the internet

In addition to interviewing twenty women, 8 stories that explained what motivated a choice to birth outside the system, were accessed from public sources including submissions to the NMR and those published on the internet. This was done firstly, in an effort to determine whether saturation of the categories was achieved. Secondly, women who *birth outside the system* are part of close-knit communities and often express similar views. Analysis of the NMR submissions was similar to the data that had already been collected and this was reassuring, and no new online stories were sought.

### Demographic information

Demographic information was collected from the 20 women who were interviewed. The median age of the women when interviewed was 34. The majority of women had their first baby within the system, with only two women birthing outside the system for their first baby. Nine out of the 20 women were employed at the time of interview. The most remarkable finding from these data was the participant group’s high level of education. Compared to the general population at the time, all participants were highly educated, with 14 out of the 20 having a bachelor degree or higher. One participant had a doctoral degree, and overall, 70% of the participants had a tertiary qualification. In 2019, 36% of Australian women between 25 and 64 years of age held a bachelor degree or higher [32]. This indicates that the participants belong to a highly educated group. It was found that all of the participants lived within a 30-min drive to a hospital that provided maternity care. It is possible, therefore, that the choice to birth outside the system was considered more reasonable given the women’s relative proximity to emergency care. Four of the twenty participants in this study were midwives; one choosing freebirth and three choosing homebirth. This adds an interesting dimension, as these women had insider knowledge of mainstream maternity services. Demographic information collected included the proximity of the place of birth to a maternity care service. This was collected to ascertain if access to hospital services was a factor in women’s decision-making process. By collecting this data we could determine that none of the research participants chose to give birth outside the system as a result of living remotely and not having access to a maternity care facility.

### Using field notes and memos

Throughout the research process, the writing of detailed field notes and memos was performed after each data collection and data analysis session. The generation of memos is considered an essential element of Grounded Theory, with memos seen to be ‘the bedrock of theory generation’ [33].

### Concurrent data collection and analysis

Grounded Theory ‘is an iterative concurrent analytical method of constantly comparing and collecting or generating data that results in high-level conceptual abstract categories…’ [28]. A fundamental principle of Grounded Theory is that data collection and data analysis occur simultaneously [27]. The researcher then compares codes and categories, thus building theory from the data itself [27]. Constant comparative analysis was aided by the use of ‘Nvivo’ [34], a data management software program that allows the researcher to group similar aspects of the data together. The purpose of data analysis in Grounded Theory is conceptual development; this is done by coding and categorising the data [27].

### Finding the Core category and basic social process

In this study the ‘core category’ explained what motivates women to give birth outside the system. It was called ‘wanting the best and safest’. Secondly, the ‘basic social process’, which explains the process of how the core category evolved was identified as ‘finding a better way’ as it explained how they came to the decision to birth outside the system. In this study, the basic social process represents the actions and journey the participants took, which ultimately led them to choose birth outside the system. The use of ‘storyline’ in the analysis of the data propelled this research into a Grounded Theory of what motivates women to give birth outside the system.

‘Storyline’ was used as a tool to overcome a period of stagnation between initial and intermediate coding before moving on to advanced coding. Strauss and Corbin (1990) define the ‘story’ as ‘a descriptive narrative about the central phenomenon of the study’ and ‘storyline’ as ‘the conceptualization of the story’ [35]. Birks and Mills (2011) suggest that not only can the use of storyline assist in the production of the final theory, but that it also enables the theory to be presented to the reader in an accessible, intelligible and palatable format [28].

### Ethical considerations

Ethics approval was obtained from the University Human Research Ethics Committee (Approval Number: H8248). Ethical conduct of a research project requires continual sensitivity of the researcher toward the participants [36], and this is something that was taken seriously throughout the entirety of this research project. Pseudonyms and changes were made to details that were potentially identifying. The participants were offered the option of ceasing the interview at any time if they were distressed and offered contact information for counseling services if required. Two participants were asked if they would like to cease the interview after distress was observed; however, in both cases, the participant requested that the interview continue.

### Reflexivity

Throughout the research process, the researchers identified how their own beliefs could impact upon the research and maintained strategies to counteract bias [29, 37]. These strategies included memo writing, frequently asking ‘what is the data saying?’ and creating spreadsheets with hard evidence from the data to prove to ourselves that what we were interpreting the data to say, was actually being said. All three authors are midwives and the first author had her own baby ‘outside the system’ towards the end of the study thus becoming an insider in this research project.

## Results

**Fig. 1:**
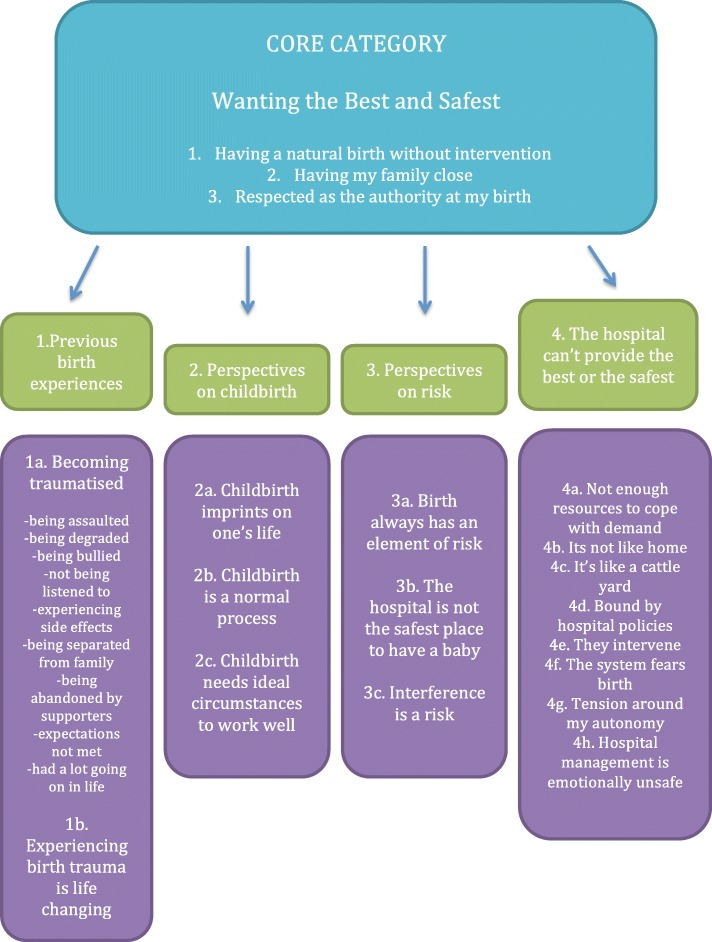
The core category

The core category (Fig. 1) that emerged was ‘wanting the best and safest,’ which describes what motivated the women’s decision to *birth outside the system;* because they believed it was the best and safest for them and their baby. How they came to this belief is explicated through the subcategories: ‘previous birth experiences,’ ‘perspectives on childbirth,’ ‘perspectives on risk’ and ‘the hospital can’t provide the best or safest.’

**Fig. 2:**
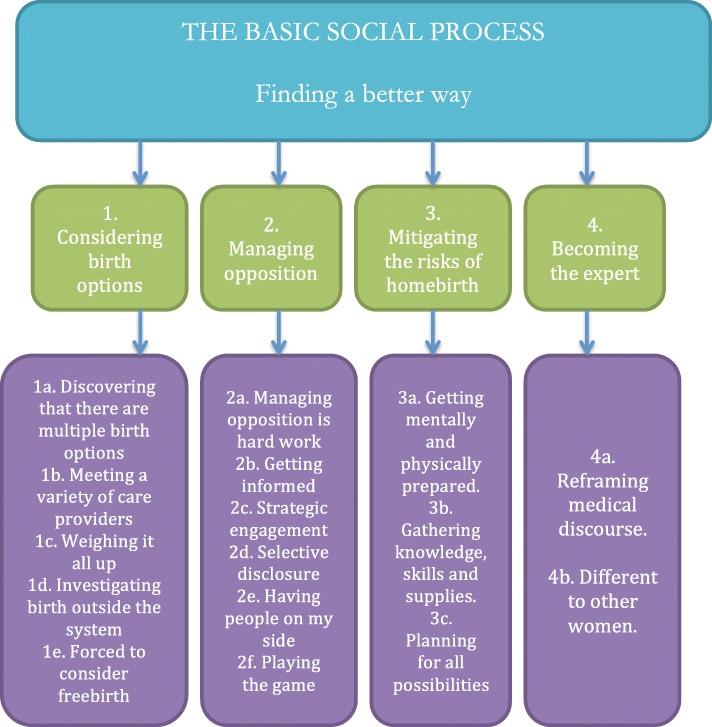
The basic social process

The basic social process (which explains the journey women took as they pursued the best and safest) was ‘finding a better way’ (Fig. 2). This process is elucidated through the subcategories: ‘considering birth options,’ ‘managing opposition,’ ‘mitigating the risks of birth at home’ and ‘becoming the expert.’

### Wanting the best and safest

This study found that what motivates women to *birth outside the system* is ‘wanting the best and safest’ for themselves and their babies. One participant who chose homebirth explained:

‘In an effort to want to make my own choices and to be in control and to feel safe, and like the decisions were truly mine and the decisions that are the best for not just you and not just your baby, not just your husband, but just the whole picture. It’s what’s best for me and this baby and my other children and my husband, it’s wanting the best and to get the best I feel like I need to be in control and for me to be in control and to be safe means I need to be at home’. (HB05).

Similarly, a woman who chose freebirth explained:

‘I want the absolute very best for all of my children, I would not ever, ever endanger them, I don’t want them harmed, I want them to have the very best outcome in terms of their physical health, mental health, emotional health, their complete and utter wellbeing and safety…so that’s where I was coming from’. (FB05).

There are three criteria by which the participants judge ‘best’ and ‘safest.’ These are: having a natural birth without intervention, having their family close and being respected as the authority throughout their care. If a birth option cannot cater to one of these criteria, it is not considered the best or the safest.

#### Previous birth experiences

The women in this study who had given birth previously described their previous birth experiences as a learning experience, which gave them insight into what they did and didn’t want for their next births, and many were also left feeling traumatised. After becoming traumatised by their previous birth experiences, the participants learnt that a birth inside the system could not offer them the standard of care they desired. One woman recounts:

'This male doctor and a nurse came in and he was saying, “I’m just going to examine you” ... I was saying “no” and he said “oh it won’t take a minute just slide back” and started, and I was like “stop it, stop it, get out of me: and he was like “just lie still,” and he ended up holding me down and a nurse held me up at the top …while he ‘examined me,’ that was so painful ... in the street, assault is assault, if you’re saying no and there’s a person still continuing doing what they are doing that is assault'. (FB01).

Another woman described her experience of caesarean section where she also felt disregarded as a human being, stripped of dignity and degraded:

'I was then treated like a piece of meat, my baby was handed to my partner and he was told to leave the theatre, I was then stripped of all coverings while the staff wandered around theatre cleaning up, I was then told that they were now going to clean my vagina, I was exposed for all to see. I was in such a state of shock by this stage that I was unable to speak, let alone object to what was happening to my body or to ask for my baby. I was stripped of all dignity and totally degraded'. (FB15).

These circumstances taught the participants that hospital birth could not offer them safety, because they had already proved psychosocially to be dangerous. For this reason, the participants perceived births inside the system to be a riskier and a less safe choice than a *birth outside the system*. This belief was accentuated by the impact birth trauma had on their lives. Some of the participants’ experience of birth trauma was life changing, an effect they were not willing to compound by returning to the place that was the source of their original trauma. So traumatic was the experience for some participants, that they reported outcomes such as the development of mental illnesses and pathological behaviors, Post Traumatic Stress Disorder (PTSD), the inability to embody the type of mothers they wanted to be, and the inability to function with their other relationships.

One woman asserted ‘that [the first] birth caused myself and my family significant distress and trauma for a long time afterwards’ (FB10). The presence of long-term effects after a traumatic birth left some participants questioning whether they would ever recover, with comments such as ‘I’ve never been the same since’ (HB11) and ‘it would change our family forever’ (FB11).

#### Perspectives on childbirth

The participants’ philosophical standpoint on childbirth played a particular role in their choice to *birth outside the system*. The participants believed that childbirth imprints on one’s life, is a normal process and needs ideal circumstances to work best. With the understanding that childbirth imprints on one’s life, participants pursued *birth outside the system* in the hope that the imprinting would be positive not negative. The belief that childbirth is a normal bodily function, predisposed participants to question the need for hospitalisation. Finally, the participants reported a detailed list of circumstances that they believe are required to ensure birth works best. These included: adequate hormonal function, an optimal environment, privacy and relaxation, active birth positioning and good physical, emotional and mental preparation. Based on their previous experiences, the participants believed that the hospital was not capable of facilitating the circumstances that birth needed to work best and was therefore incapable of providing the best and safest.

#### Perspectives on risk

Just as the participants’ perspectives on childbirth informed their choice to *birth outside the system*, so too did their perspectives on risk. The participants’ perceptions of risk were that birth always entails an element of risk, the hospital is not the safest place to have a baby, and that interference is a risk. The participants also believed that their chosen place of birth may not mitigate the risk inherent in birth. As one woman put it,

‘I always knew that there was no guarantee that the baby would be born alive or that it would live beyond the birth, but I think there is no guarantee with that in a hospital setting either’. (FB06).

They believed that the hospital is not the safest place to have a baby, citing additional and unique risks associated with this option. One woman commented:

‘Automatically walking into a hospital I’m exposed to hospital bugs, that to me is unsafe ... a neonate’s immune system is not fully developed, I don’t want my babies exposed to that, I don’t even want myself exposed to that. So, they can’t possibly offer me a safe birth’. (FB05).

Finally, the participants perceived that interference in the birth process is a risk. One woman commented:

'If you can stay away from the hospital system, then you can minimise the amount of interference. I look at interference a bit like risk, like every time someone new comes across you or does something that’s a risk that something goes wrong, every time you get a medication there is a risk it’s the wrong one, every time they do something, there is a risk that flows onto something else, so if no one is doing anything to you or giving you any drugs or performing any unnecessary tests, then there is no risk there'. (HB05).

Because the participants wanted the best and safest for themselves and their babies, they sought out birth options that would limit risk. Hospital was perceived to be riskier, and the most likely site where birth would be interfered with, so it was discounted as a suitable birth option.

#### The hospital can’t provide the best or safest

Participants’ previous experiences with the hospital system, paired with their beliefs on childbirth and risk, led them to assume that the hospital could not provide the best or the safest, because they could not cater to their personal criteria by which ‘safest’ and ‘best’ were judged. The participants cited multiple reasons why the hospital was incapable of providing the best or safest and these have been categorised under the headings, ‘not enough resources to cope with demand’, ‘the environment was not like home’, ‘it’s like a cattle yard’, ‘staff are bound by hospital policy’, ‘they intervene’, ‘they fear birth’, ‘there would be tension around my autonomy’ and ‘hospital management of birth is emotionally unsafe’.

One woman felt that the hospital system was inherently flawed, stating:

'I’m not sure they really could have done anything better for me just because of the mentality ... it’s a revolving door and they’ve got to get this baby out the quickest way possible, whichever way suits us and then get this baby fed, don’t care how just get it fed and then get you out the door. I don’t know if they could have done anything better'. (HB07).

Worse still, many women found hospital care traumatising, with one woman explaining,

'I decided that should I find myself unable to access a midwife, I would birth at home – alone. Nothing that can happen to me or my baby at home could be much worse than what my second baby and I experienced in hospital. I will never subject myself, my baby or my family to such an ugly, traumatic and dehumanising experience again'. (FB1).

The participants concluded that the hospital could not provide the best or the safest, and so they set out on the journey towards ‘finding a better way’, which ultimately led them to *birth outside the system*.

### The basic social process- finding a better way

In explaining their choice to *birth outside the system*, the participants described making a considered decision after first exploring other birthing options. This process followed a typical path, with the women ‘discovering that there are multiple birth options’. They move on to ‘meet with a variety of care providers’ and then ‘weigh it all up.’ After making the decision to circumvent the system, the women ‘get informed about out-of-the-system birthing options,’ which for some leads to homebirth and for others ‘forces them to consider freebirth.’

#### Considering birth options

As the women investigated, they discovered many birthing options of which they had hitherto been unaware. In her pursuit of a better way, one participant said,

‘I just became more informed about my other choices ... [and this] just blew open a whole new world for me around another choice’ (HB04).

As the women met with care providers and weighed up all their options, they came to a realisation that giving birth in hospital would not cater to their desire for the best and safest. It was from this point women described immersing themselves in information about homebirth and freebirth; one woman explains:

‘I googled everything and anything that I could get my hands on…read Ina May Gaskins stuff and yeah so got a lot more informed about the alternatives’ (HB06).

For the women who chose homebirth with risk factors, their process of discovery of birth options stopped here; they hired a midwife and followed through with their midwife-assisted homebirth. For those who chose freebirth, the process of considering their options continued. In their quest to find a better way, some participants came to point where they were forced to consider freebirth. This was because other birthing options became either unavailable or unacceptable to them.

All but one of the women reported having made contact with a midwife or care provider in order to discuss their birth options. The one woman who did not make contact had completed a PhD on the topic of medicalisation of childbirth and during that process had connected with women who described their choice to freebirth. Prior to becoming pregnant, this woman decided she would freebirth and thus did not engage the counsel of a care provider in the pursuit of her plans to freebirth.

While the women choosing homebirth with risk factors chose to continue being cared for by a midwife and women who chose freebirth disengaged from this care, the two choices to give birth outside the system have their roots in a fundamental rejection of how maternity care is provided within the system. These two outside-the-system birth choices are united in their counter-cultural rejection of the care that is offered to women in mainstream maternity care services in Australia.

The majority of participants reported a preference to have a midwife in attendance, but ultimately freebirthed because they felt they had no other option, and so it was the best and safest option available to them at the time. One participant explained:

'Yeah, like, if that [a midwife] had been available, I would have been quite happy to have the midwife help me in my home have my baby, I never would have considered unassisted ... I mean like I said, I never would have chosen to go down that path had the decision – I kind of felt like the decision was made for me, by denying me that choice'. (FB03).

#### Managing opposition

In the process of finding a better way to birth, the participants realised that their chosen option subverted biomedical models of childbirth, and that they would have to formulate a strategy in order to manage opposition. The women anticipated that managing opposition within a hospital setting would be hard work, and they did not want to have face this task while in labour. As one woman put it, ‘I felt like it would be a constant struggle, my partner and I against the hospital staff’ (HB04). Another participant felt that managing opposition ‘seems like a lot of energy’ (HB06) to waste whilst trying to give birth.

The strategies employed in order to manage perceived opposition included: arming themselves with knowledge so they could effectively defend their choice, strategic engagement of care providers – avoiding those who would oppose their choice and engaging those who would facilitate it, selective disclosure of their plans to avoid conflict, having people on their side to help advocate for their choice, and ‘playing the game,’ which involved bartering with and manipulating the system to ultimately get what they wanted without having to compromise on the best and safest.

#### Mitigating the risks of birth at home

The participants made their birth choices based on their desire for risk reduction, therefore, they sought to mitigate the risk by focusing on their mental and physical preparation in order to experience the most optimal birth outcomes. One woman explained:

'I really feel like setting the scene for the freebirth for me was all about taking good care of myself and I invest a lot of time and money into having really good health care and I take really good care of myself'. (FB06).

They also gathered knowledge, skills and supplies to ensure that they felt adequately prepared to mitigate their unique risks, this preparation differed between women who chose freebirth and homebirth. The women who chose freebirth read about what they might need – as one participant noted, ‘we did a lot of research into what we would need to have the birth at home’ (FB01). Women who freebirthed collected equipment for resuscitation – ‘we had the little resuscitation kit’ (FB01) – and also equipment that would be required for an uncomplicated birth, ‘like sterilised scissors’ (FB05) to cut the cord after the birth. While, women who had hired a midwife did not make mention of specific items that they gathered in preparation for their homebirth, the women who freebirthed felt obliged to gather this equipment since they were taking full responsibility over what supplies would be available to them for their birth.

They also planned for all possibilities, so that they were clear in their minds about what plan of action would be taken in an unexpected circumstance. In the circumstance of an emergency, the participants believed that transferring to hospital became the new best and safest. One participant had prepared for:

'... everything from, if my waters break and there is staining in the meconium we are off to hospital, if you know, if I’m feeling unwell – you know we went through a – I listed all the situations with my husband and I sort of said if this happens, then we need to transfer to hospital, if that happens then we need to transfer to hospital'. (FB08).

#### Becoming the expert

Throughout the process of finding a better way, the participants simultaneously became the experts. They came to value their own ability to make informed and safe choices over that of their care providers. One woman described:

'I felt empowered to be able to take a certain amount of control over my own care ... I think really largely for me, it’s really been a progressive experience of feeling more confident the more skilled up as time passed'. (FB06).

Another woman spoke of how her previous birth experiences had turned her into an expert. Having gained confidence and expertise through her previous birth experiences, she felt confident to birth her breech baby at home:

'It was my third birth. I think that is a big factor. If it was my first birth, I probably would have listened to the obstetrician and just gone for the elective caesarean out of fear, so again I was very comfortable with birthing babies by this stage, very confident in my own ability'. (HB06).

The participants described themselves as different to other women because they are ‘always bucking against the system’, ‘take responsibility’, ‘investigate to ensure that they know’, ‘believe in their ability to ‘know,” ‘have a sense of entitlement to choose’, and ‘possess confidence in their ability to birth’. These characteristics facilitated the development of their expertise as they pursued a better way and ultimately led them to *birth outside the system*.

## Discussion

This Grounded Theory study aimed to explore what motivates women to *birth outside the system*. In this study *Birth outside the system* was considered to be the best and safest option compared to other birthing options available. These findings bring into question the authority of biomedical ways of knowing and managing birth.

### Authoritative knowledge

Women who choose to give birth outside the system hold the belief that they, rather than the socially defined birth experts, hold the authority to define risk and safety. Authoritative knowledge describes the knowledge that ‘counts’ in a particular situation, is the basis on which decisions can be made, and provides justification for a course of action [38]. Authoritative knowledge is subjective and not necessarily correct but holds authoritative power; Its power is not that its ‘right’, but that it counts [38]. The women who chose birth outside the system buck against the authoritative knowledge of health practitioners and trust their own intuitive and cognitive knowledge. This diminishes their reliance upon practitioners and increases their responsibility over monitoring the well-being of themselves and their babies. At its core, authoritative knowledge deligitimises other knowledge [39], so if care providers claim they have authoritative knowledge, they are simultaneously claiming that other knowledge doesn’t equate to their own. Giving birth outside the system, discounts the authority of medical care providers to know and define risk and safety in birth and puts the authority and also the responsibility into the hands of the woman.

### Interpretations of safety

Women seek to give birth where they feel safe and make decisions that prioritise the health and well-being of their baby [40]. However, beliefs about safety differ vastly [41] and the participants’ concept of safety accorded equal importance to their physical, emotional, social, spiritual, cultural and psychological safety. This valuing of multiple aspects of safety comes into conflict with biomedical models of care [42] which equates reducing physical risks with increasing safety [43].

The rhetoric of biomedical birth practices argues that interventions are employed in order to enhance safety [21, 44]. However, interventions create iatrogenic risk [45], and sometimes women sustain psychological and emotional trauma as a result [46]. Critics suggest that intervening in birth can cause more problems than it solves, increasing the complexity of birth beyond what would otherwise occur [47]. This was the experience of the women in this study, who attributed the treatment they received in hospital with causing complications and trauma. Thus, the participants questioned and challenged the premise that giving birth in hospital equates to safety and cognitively calculated that greater safety would be offered to them in the home environment.

### Risk and risk management in childbirth

Current risk discourse dictates that giving birth is risky and hospitals are the safest place to give birth. However, women who gave birth outside the system believed that, although childbirth carries an element of risk, that a hospital is not the safest place, or an effective way to mitigate risk. Bisits (2016) suggests that the risks associated with pregnancy and birth are low prevalence phenomena, however there is an obstetric tendency towards an emphasis on risk. He suggests that when presented with the range of ways pregnancy and birth can go wrong, women begin to feel that pregnancy is dangerous [48]. Perpetuating fear by promoting risk is an effective strategy to position hospitals as the safest place to give birth; it allows the birthing community to exist in a heightened sense of alarm and thus increases their willingness to attend hospitals [49]. Scamell calls this the ‘scare factor’ [50] and, De Vries (2010) argues medical professionals gain control by inventing risk [51].

It was apparent in this study that women considered risk seriously but placed the iatrogenic risks of giving birth in a hospital under intense scrutiny, challenging implicitly agreed assumptions that hospital birth must be safer and exposing risks that are often simply accepted as part of birth [17]. In this study, the participants became their own expert in their pursuit of a better way to birth. This signified the taking of responsibility over the outcome of their choices. The women in this study rejected the obstetric claim of responsibility over the defining and management of risk in birth. In so doing, they reclaimed jurisdiction over risk management and the subsequent outcomes of their pregnancy and birth. Women who birth outside the system did not accept that a hospital is the best place to mitigate the risks inherent in birth. Therefore, they did not see the need to birth in hospital in the absence of actual complications.

### Trauma in childbirth

Globally, bewteen 20–48% of women are reporting their birth expereince as traumatic [52]. Similarly, the majority of the participants in this study perceived their previous birth expereinces to be emotionally, mentally, socially, culturally and physically unsafe and as a result, traumatizing. For these women, the choice to *birth outside the system* came from a desire to prevent a repeat of past traumatic events.

The body of literature about the role of care providers in birth trauma is growing and shines the spotlight on maternity care providers as the instigators of events that have left women feeling traumatised [52, 53]. It has been suggested that when women have clear birth preferences, they may be more prone to trauma [54]. Suggestions like these focus on the predisposition of the woman to having feelings of trauma. However, what current evidence is demonstrating is that care provider actions and interactions can negatively influence women’s experience of birth [55] and shifts the supposed blame onto the care providers rather than the woman. Poor quality interactions with care providers have been identified as a major risk factor for women who describe being traumatised by their birth experience [52]. Harris and Ayres (2012) found that the main incidents that caused birth related PTSD were ‘interpersonal difficulties’ and ‘being ignored’ [56]. Women also identified lack of support, poor communication, being abandoned and being put under pressure as circumstances that contributed to their birth trauma [56]. Beck (2004) reports participants feeling raped, abandoned and stripped of dignity by their care providers and the women in this study also went into detail about non-consensual procedures [57]. This non-consensual behavior is also highlighted in more recent research where women described being held down by staff while having interventions such as vaginal exams were forced upon them [55]. What is becoming obvious is that emotional trauma in childbirth is developing as an iatrogenic consequence of standard maternity care in hospitals.

### Maternal control, choice and autonomy

The difference between a biomedical and humanised approach to maternity care is whether or not the woman giving birth retains control [58]. The struggle between biomedical and social birth philosophies centers on who should control what happens in childbirth. Meyer (2012) identified four attributes of control: having a lead role in decision-making, having access to information, the feeling of personal security through a humanised approach to care and, having control over their physical functioning, such as what would happen to their bodies [59]. The participants in this study desired ‘external control,’ which denotes control of the environment, procedures, the actions of care providers and the decision-making processes that take place [60]. However, Edwards (2013) rightly comments that choice within a hospital system is limited by a ‘predetermined obstetric menu’ (p. 214) so if women desire something that is not on the ‘menu’ they must seek alternative birth options [61].

The women in this study demonstrated that their concept of safety included ‘being the authority at my birth,’ which meant being able to control their birthing circumstances. The feeling of being in control is a significant factor in women perceiving their birth experience as satisfying and positive [59]. A common cultural misconception is that women actually do have control over what is done to them in hospital. Lothian notes, however, that ‘[i]t’s an illusion. No matter what anybody tells you… the bottom line is, you will follow the rules of the hospital, and you will do what your doctor wants you to do’ [62]. This aligns with the critiques of the maternity care system offered by the participants in this study, who chose to *birth outside the system* in order to preserve their autonomy. Similarly, Wagner (2001) suggests that if a woman wishes to control and humanise her birth, she must move outside of the hospital [58].

#### Limitations of this research

The limitations of this research include that it was undertaken in Australia so may not be generalisable to other countries. Aboriginal and Torres Strait Islander women were not interviewed for this study and are a group known to avoid mainstream services in Australia. In addition, the study group was not culturally diverse, with the majority being white Caucasian, socially advantaged women. Women who chose to *birth outside the system* and experienced poor perinatal outcomes were not included in this study. As all the participants had a live and overall healthy baby, this study is unable to provide insight into the thoughts of women who experienced adverse outcomes and how they feel about their birth choice after the fact, though other researchers have begun addressing this [63] .

#### Recommendations

Shortfalls in the Australian maternity care system were major contributing factors to women’s choice to give birth outside the system. If the Australian maternity system neglects to provide evidence-based care and limits birthing options, women will continue to birth outside the system. Improvements to maternity policies and practice should prioritise humanising maternity care by providing continuity of midwifery care models for all women including women with complex pregnancies, one-to-one midwifery care during birth, woman-centered care, and midwifery-led care in public homebirth programs and standalone birth centres. Finally, enhancing support for private midwifery services would significantly reduce the number of women opting for freebirth as, based on this research, women would be more likely to access a midwife in order to birth at home over choosing freebirth. Perhaps if women felt less disillusioned by their maternity care options and they felt safe within current maternity care options, they would not be forced to consider birth outside the system [5, 6]. Adopting maternity care strategies that allow for women who decline medical care and wish to avoid routine, policy-based care could also encourage women to engage with the maternity care system.

## Conclusion

This Grounded Theory study aimed to explore what motivates women to *birth outside the system*. In this study *Birth outside the system* was considered to be the best and safest option compared to other birthing options available. Shortfalls experienced within the Australian maternity care system were major contributing factors to women’s choice to *birth outside the system*.

## Data Availability

We cannot share this data as it is highly controversial, sensitive and we guaranteed anonymity. The qualitative data could be identifiable.

## Abbreviations

BBA: Born before arrival
NMR: National review of maternity services in Australia
FB: Freebirth
HB: Homebirth
PTS: Post traumatic stress disorder

## References

[1] Welfare AIoHa. Australia’s mothers and babies 2015-in breif. Perinatal statistics series no.33. Cat no. PER 91. Canberra: Canberra Australian Institute of Health and Welfare; 2017.

[2] Thornton CE, Dahlen HG. Born before arrival in NSW, Australia (2000–2011): a linked population data study of incidence, location, associated factors and maternal and neonatal outcomes. Australia: BMJ Open. 2018;8(3):1–8. 10.1136/bmjopen-2017-019328 PMC5857678 29540412

[3] Newman LA (2008). Why planned attended homebirth should be more widely supported in Australia. Aus N Z J Obstet Gynaecol.

[4] Hollander M, de Miranda E, van Dillen J, de Graaf I, Vandenbussche F, Holten L. Women's motivations for choosing a high risk birth setting against medical advice in the Netherlands: a qualitative analysis. BMC Pregnancy Childbirth. 2017;17(1):1–13. 10.1186/s12884-017-1621-0 PMC5732454 29246129

[5] Feeley C, Thomson G. Why do some women choose to freebirth in the UK? An interpretative phenomenological study. BMC Pregnancy Childbirth. 2016;16(1):1–12. 10.1186/s12884-016-0847-6 PMC4802706 27000100

[6] Feeley C, Thomson G (2016). Tensions and conflicts in ‘choice’: Womens’ experiences of freebirthing in the UK. Midwifery..

[7] Feely C, Burns E, Adams E, Thomson G. Why do some women choose to freebirth? A meta-thematic synthesis, part one. (Report).Evidence Based Midwifery. 2015;13(1):4.

[8] Rigg E, Schmied V, Peters K, Dahlen H (2015). Not addressing the root cause: an analysis of submissions made to the south Australian government on a proposal to protect midwifery practice. Women Birth..

[9] Holten L, de Miranda E (2016). Women′s motivations for having unassisted childbirth or high-risk homebirth: an exploration of the literature on ‘birthing outside the system’. Midwifery..

[10] Lindgren HE, Nässén K, Lundgren I (2017). Taking the matter into one's own hands – Women's experiences of unassisted homebirths in Sweden. Sex Reprod Healthc.

[11] Oboyle C (2016). Deliberately unassisted birth in Ireland: understanding choice in Irish maternity services. Br J Midwifery.

[12] Plested M, Kirkham M (2016). Risk and fear in the lived experience of birth without a midwife. Midwifery..

[13] Rigg E, Schmied V, Peters K, Dahlen HG. Why do women choose an unregulated birth worker to birth at home in Australia: a qualitative study.(Report). BMC Pregnancy Childbirth. 2017;17(1). 10.1186/s12884-017-1281-0 PMC5371179 28351344

[14] Symon A, Winter C, Donnan PT, Kirkham M (2010). Examining Autonomy’s boundaries: a follow-up review of perinatal mortality cases in UK independent midwifery. Birth..

[15] Feeley C, Burns E, Adams E, Thomson G (2015). Why do some women choose to freebirth? A meta-thematic synthesis, part one. Evid Based Midwifery.

[16] Miller AC (2009). “Midwife to myself”: birth narratives among women choosing unassisted homebirth*. Sociol Inq.

[17] Jackson M, Dahlen H, Schmied V (2012). Birthing outside the system: perceptions of risk amongst Australian women who have freebirths and high risk homebirths. Midwifery..

[18] Dahlen H, Schmied V, Tracy SK, Jackson M, Cummings J, Priddis H (2011). Home birth and the National Australian Maternity Services Review: too hot to handle?. Women Birth..

[19] Dahlen H, Kumar-Hazard B, Schmied V (2020). Birthing outside the system: the canary in the coal mine.

[20] Donnellan-Fernandez R. Having a Baby in Australia: Women's Business, Risky Business, or Big Business? Outskirts. 2011;24 N_A.

[21] Murphy-Lawless J (1998). Obstetrics & Gynaecology Journal.

[22] AMA. (2010). New Study confirms high risk of homebirths. Retrieved 11-09-2012, from http://ama.com.au/node/5273.

[23] Hutton E, Reitsman A, Simioni J, Brunton G, Kaufman K. Perinatal or neonatal mortality among women who intend at the onset of labour to give birth at home compared to women of low obstetrical risk who intend to give birth in hospital: a systematic review and meta-analyses. EClinicalMed (lancet). 2019; in press, Corrected Proof (https://www.sciencedirect.com/science/article/pii/S2589537019301191#!). 10.1016/j.eclinm.2019.07.005 PMC6833447 31709403

[24] Scarf VL, Rossiter C, Vedam S, Dahlen HG, Ellwood D, Forster D, Foureur MJ (2018). Maternal and perinatal outcomes by planned place of birth among women with low-risk pregnancies in high-income countries: a systematic review and meta-analysis. Midwifery..

[25] Homer CSE, Cheah SL, Rossiter C, Dahlen HG, Ellwood D, Foureur MJ, et al. Maternal and perinatal outcomes by planned place of birth in Australia 2000–2012: a linked population data study. BMJ Open. 2019;9(10):240–55. 10.1136/bmjopen-2019-029192 PMC6830673 31662359

[26] Dahlen HG, Jackson M, Stevens J (2011). Homebirth, freebirth and doulas: casualty and consequences of a broken maternity system. Women Birth..

[27] Denzin NK, Lincoln YS (2005). The sage handbook of qualitative research.

[28] Birks M, Mills J (2011). Grounded theory: a practical guide.

[29] Charmaz K (2006). Constructing grounded theory: a practical guide through qualitative analysis.

[30] Glaser BG (1992). Emergence vs forcing: the basics of grounded theory analysis.

[31] Co A (2009). Improving maternity services in Australia, report of the maternity services review Canberra: commonwealth of Australia.

[32] A.B.S. Australian Social Trends. Australian Bureau of Statistics 2011.

[33] Glaser BG. Theoretical sensitivity: advances in the methodology of grounded theory. Mill Valley: Sociology Press; 1978.

[34] NVIVO. NVIVO qualitative data analysis software, version 9: QSR International; 2010.

[35] Strauss A, Corbin J (1990). Qualitative analysis for social scientists.

[36] Marshall C, Rossman GB (2011). Designing qualitative research.

[37] Clark A (2005). Situational analysis: grounded theory after the postmodern turn.

[38] Jordan B, Davis-Floyd RE, Sargent CF (1997). Authoritiative knowledge and its construction. Childbirth and Authoritative Knowledge: Cross-cultural perspectives.

[39] D. Bonaparte A (2015). Physicians' discourse for establishing authoritative knowledge in birthing work and reducing the presence of the granny midwife. J Hist Sociol.

[40] Fisher C, Hauck Y, Fenwick J (2006). How social context impacts on women's fears of childbirth: a Western Australian example. Soc Sci Med.

[41] Capelli I (2011). Risk and safety in context: medical pluralism and agency in childbirth in an eastern Moroccan oasis. Midwifery..

[42] Lothian J (2012). Risk, safety, and choice in childbirth. J Perinat Educ.

[43] Edwards N (1997). Women's Decision-making Around Home Birth. AIMS J.

[44] Davis-Floyd RE (1992). Birth as an American rite of passage.

[45] Healy S, Humphreys E, Kennedy C (2017). A qualitative exploration of how midwives’ and obstetricians’ perception of risk affects care practices for low-risk women and normal birth. Women Birth..

[46] Kitzinger S (2006). Birth crisis.

[47] Davis-Floyd RE, Barclay LM, Daviss B, Tritten J (2009). Birth models that work.

[48] Bisits A (2016). Risk in obstetrics – perspectives and reflections. Midwifery..

[49] Dahlen HG (2016). The politicisation of risk. Midwifery..

[50] Scamell M (2016). The fear factor of risk – clinical governance and midwifery talk and practice in the UK. Midwifery..

[51] De Vries C (2010). Birth in an ordinary instant. J Perinat Educ.

[52] Simpson M, Schmied V, Dickson C, Dahlen HG (2018). Postnatal post-traumatic stress: an integrative review. Women Birth.

[53] Priddis HS, Keedle H, Dahlen H (2018). The perfect storm of trauma: the experiences of women who have experienced birth trauma and subsequently accessed residential parenting services in Australia. Women Birth..

[54] Goodman P, Mackey MC, Tavakoli AS (2004). Factors related to childbirth satisfaction. J Adv Nurs.

[55] Reed R, Sharman R, Inglis C. Women’s descriptions of childbirth trauma relating to care provider actions and interactions. BMC Pregnancy Childbirth. 2017;17(21). 10.1186/s12884-016-1197-0 PMC5223347 28068932

[56] Harris R, Ayers S (2012). What makes labour and birth traumatic? A survey of intrapartum 'hotspots'. Psychol Health.

[57] Beck TC (2004). Birth trauma: in the eye of the beholder. Nurs Res.

[58] Wagner M (2001). Fish can't see water: the need to humanize birth. Int J Gynecol Obstet.

[59] Meyer S (2013). Control in childbirth: a concept analysis and synthesis. J Adv Nurs.

[60] Vandevusse L (1999). Decision making in analysis of women’s birth stories. Birth..

[61] Keedle H, Schmied V, Burns E, Dahlen HG (2019). A narrative analysis of women's experiences of planning a vaginal birth after caesarean (VBAC) in Australia using critical feminist theory. BMC Pregnancy Childbirth.

[62] Lothian JA (2008). The journey of becoming a mother. J Perinat Educ..

[63] Rigg EC, Schmied V, Peters K, Dahlen HG. A survey of women in Australia who choose the care of unregulated birthworkers for a birth at home. Women Birth. 2018;33(1):86–96. 10.1016/j.wombi.2018.11.007 30503223

